# Supplementary material for: Impact of gaseous ozone treatment of fish carcasses (Gadus morhua) on the microbiological load and their quality
Source: PLoS One. 2025 Aug 18;20(8):e0327866. doi: 10.1371/journal.pone.0327866 (PMC12360513; doi:10.1371/journal.pone.0327866)
Supplement: Attachment — Submitted filename: Response for Reviewers.docx [file pone.0327866.s005.docx]

Reviewer #1: Summary and General Comments

The manuscript explores the effect of ozone treatment on microbial load and chemical quality parameters of fish carcasses. The study's aim—to assess the balance between microbial safety improvement and chemical stability of fish quality under ozone exposure—has potential relevance in food preservation research. However, the study suffers from several critical flaws that affect the validity and impact of its findings. Specifically, the manuscript lacks rigor in methodological design, clarity in data interpretation, and provides insufficient discussion on the broader implications of the results.

Major Concerns
Experimental Design and Controls:

The study's methodological approach to control sample treatment lacks detail, specifically regarding storage conditions and any baseline tests performed. Further, the exposure times to ozone (10, 20, and 30 minutes) do not account for varying ozone concentrations, leaving questions about the applicability of these results under different conditions.
The authors do not describe the basis for selecting 20 ppm as the ozone concentration or how this relates to commonly accepted or regulatory levels for ozone exposure in food.

Action taken:

In section “determination of microbial load” a comment was added:

The ozone dose can be modified by changing in the exposition time at the same ozone concentration or by changing the ozone concentration at the same exposition time In this study, the ozone dose was controlled by modifying the exposition time at constant ozone concentrations. To analyze the efficacy of ozonation treatment and examine the complex effects accompanying ozonation, the fish samples were exposed to ozone within three different time intervals at a concentration of 20 ppm. This concentration level was found to be sufficient to reduce the microbial load in previous studies, e.g, Antos et al., 2020.

Data Analysis and Statistical Interpretation:
The statistical analysis is inadequately explained. The manuscript lacks an account of whether differences in microbial counts and quality parameters between time points are statistically significant. For instance, the authors state reductions in microbial load but do not clarify if these reductions are meaningful or consistent across replicates.
There is also insufficient discussion on the standard deviations or confidence intervals, which would strengthen the reliability of the results presented.

Action taken:

[Additional](https://prz1.sharepoint.com/sites/ZC/Shared%20Documents/PKA%20-%20Logistyka/Aparatura/Aparatura.docx?web=1) section statistical analysis was added:

“The obtained results were analysed using STATISTICA 13.1 software (StatSoft, Palo Alto, CA, USA). One-way analysis of variance (ANOVA) was applied (α = 0.05) to show the differences between observed results.

In section “Determination of microbial load” an information on the SD values for the determined microbial loads was added.

“The SD for the observed means of microorganism count before and after ozone exposure of fish was +/- 15% e.g., between 2.7*10^2^ and 1.75*10^2^“

In section “Antioxidant potential of ozonated fish” a comment was added:

The Anova method was used for statistical analysis. The values obtained, which are marked in Fig. 1 with the same letters, are not statistically significantly different at the significance level of 0.05.

In section “Metabolites of lipids oxidation” comment have been added:

The Anova method was used for the statistical analysis; the values obtained are presented in Fig. 2 in the similar way as in Fig. 1 Significant differences between the results are indicated by different letters; significant differences are defined by the criterion α < 0.05.

Also the relevant figures were improved by addition of significance letters.

Impact on Quality Parameters and Lack of Comprehensive Discussion: The manuscript mentions the effect of ozone on lipid oxidation by tracking malondialdehyde levels and changes in the fatty acid profile, yet the discussion is brief and fails to integrate these results into a broader context. For example, the implications of increased oxidation on fish quality and nutritional value are not thoroughly discussed.

Action taken: in the introduction section a comment was added:

Although there is a great deal of data on ozone treatment in regard to microbial safety, there is a lack of data on the complex effects of the interaction of ozone with fish products. In particular, there is a literature gap on the effect of ozonation on the chemical composition of fish as well as other determinants of fish quality.

The volatile profile analysis lacks depth, particularly regarding how the detected compounds impact consumer perception and shelf life.

Action taken:

In section “Sensory analysis” a proper comment was added:

A preliminary sensory analysis of the fish tissue performed without heat treatment indicated the presence of foreign odour notes, which are typical of carbonyl products of fatty acid decomposition. Furthermore, in the volatile fraction, a representative of these compounds was identified, i.e., reported above pentadecanal (see Table 2). Due to its high volatility, this compound will not have a significant impact on the perception of consumers of heat treated products.

In section “Conclusion” a proper comment was added:

The use of the ozone procedure resulted in a statistically significant reduction of the microbial load expressed as the amount of colony forming units on the fish matrix This indicates, a potential for extension of shelf life of fish products.

The authors should compare the sensory characteristics of treated fish to typical quality standards.

The response for this comment is given above.

Literature Review and Contextualization:
The literature review is somewhat outdated, and the authors miss recent advancements in non-thermal preservation methods that could provide context for their findings.

Action taken:

The literature was updated in the introduction section by addition some recent positions (Gouvea et al., 2023; Nie et al., 2022)

There is also insufficient discussion of similar studies on ozone treatment of fish or other perishable products.

Response:

There is a literature gap on the subject of complex effects of ozonation treatment. In particular, only microbial load effect is investigated, while effects like changes in fatty acid profile or volatile faction composition were not the subject of any of known literature positions available.

Action taken:

In the introduction section a proper comment was added:

“Although there is a great deal of data on ozone treatment in regard to microbial safety, there is a lack of data on the complex effects of the interaction of ozone with fish products. In particular, there is a literature gap on the effect of ozonation on the chemical composition of fish as well as other determinants of fish quality.”

Language and Clarity Issues:
The manuscript requires extensive language revision to improve clarity and readability. Many sections contain ambiguous statements, grammar errors, and redundancies, which hinder the reader’s ability to understand the study's findings and implications.

Action taken:

Manuscript language was improved throughout the manuscript.

Minor Concerns
Figures and Tables:

Some figures, such as those showing the malondialdehyde levels and microbial load, are not labeled clearly, and it is difficult to interpret their significance without adequate legends and discussion.

Action taken:

The figures quality and their description were improved.

Table 2, which presents the volatile fraction, lacks proper labeling of columns and rows, making it challenging to understand the changes in volatile compounds.

Action taken: Table 2 was improved.

Reviewer #2: This manuscript is about the Impact of gaseous ozone treatment of fish carcasses (Gadus morhua) on the microbiological load and their quality. My recommendation on submitting the manuscript to PLOS ONE is acceptation after major revision. Please see detailed comments and questions as below

-In Title, Scientific name (Gadus morhua) should be written in italic format.

Action taken: done

-In the abstract, it is better to mention the values of the results obtained.

Action taken: abstract section was improved by the addition of microbial load values

For example, for the control sample with 1.8 x 10^3^ cfu, which was exposed to 20 ppm ozone in atmosphere during the exposition time in the range 10-30 min, a microbial load was reduced to a level between 1.6 x 10^3^ and 1.2 x 10^3^ cfu. The observed reduction levels indicated that such an ozone treatment procedure can be declared as an viable option for improvement of microbial safety of fish

- At the end of abstract, the keywords starts with first capital letter.

Action taken: keywords were corrected

-The introduction section is long and needs to be rewritten.

Action taken: the introduction section was significantly shortened.

-Due to the effect of ozone on food fat and the creation of an undesirable taste, It is better that sensory evaluation performed on the samples.

Action taken:

In section “Sensory analysis” a proper comment was added:

A preliminary sensory analysis of the fish tissue performed without heat treatment indicated the presence of foreign odour notes, which are typical of carbonyl products of fatty acid decomposition. Furthermore, in the volatile fraction, a representative of these compounds was identified, i.e., reported above pentadecanal (see Table 2). Due to its high volatility, this compound will not have a significant impact on the perception of consumers of heat treated products.

-Comparison of results with those of other similar studies, as well as discussion in the results section, is weak.

Response:

There is a literature gap on the subject of complex effects of ozonation treatment. In particular, only microbial load effect is investigated, while effects like changes in fatty acid profile or volatile faction composition were not the subject of any of known literature positions available.

Action taken:

In the introduction section a proper comment was added:

“Although there is a great deal of data on ozone treatment in regard to microbial safety, there is a lack of data on the complex effects of the interaction of ozone with fish products. In particular, there is a literature gap on the effect of ozonation on the chemical composition of fish as well as other determinants of fish quality.”

-Please insert significant letters above the columns in figures.

Action taken: significant letters were added in figures
