# Supplementary material for: Impact of gaseous ozone treatment of fish carcasses (Gadus morhua) on the microbiological load and their quality
Source: PLoS One. 2025 Aug 18;20(8):e0327866. doi: 10.1371/journal.pone.0327866 (PMC12360513; doi:10.1371/journal.pone.0327866)
Supplement: Attachment — Submitted filename: response to the reviewer PONE-D-24-48297R1.docx [file pone.0327866.s006.docx]

Note from Editor:

Note from the Editorial Office: We note that you have included the results of a new sensory analysis in your manuscript, but have not reported the details of how it was performed in your Methods section. Please include a detailed description of the sensory analysis in your Methods.

Additionally, if your sensory analysis involved human participants, PLOS One requires authors to confirm that this specific study was reviewed and approved by an institutional review board (ethics committee) before the study began. Please provide the specific name of the ethics committee/IRB that approved your study, or explain why you did not seek approval in this case. Once you have amended this/these statement(s) in the Methods section of the manuscript, please add the same text to the “Ethics Statement” field of the submission form (via “Edit Submission”).

For additional information about PLOS ONE ethical requirements for human subjects research, please refer to <http://journals.plos.org/plosone/s/submission-guidelines#loc-human-subjects-research>.

Action taken

The description of sensory analysis has been improved in the methods section:

**Method of sensory analysis**

As a part of the conducted research, the chemical investigations were supplemented only by a preliminary sensory analysis. For this purpose, the samples that were intended to be subjected to the SPME analysis were previously subjected to a preliminary sensory analysis by the authors of the manuscript, who described the sensory experiences while smelling the sample. The authors were trained to be able to participate in sensory analysis of food products and therefore also ozonated fish matrix. All of them volunteered as the subjects for sensory analysis of fish matrix exposed to ozonation. Therefore, no additional approval of ethic committee was asked for.

The discussion on sensory analysis was updated:

**Discussion on sensory analysis**

Finally a preliminary sensory analysis of the fish tissue performed without heat treatment indicated the presence of foreign odour notes, which are typical of carbonyl products of fatty acid decomposition. Furthermore, in the volatile fraction, a representative of these compounds was identified, i.e., reported above pentadecanal (see section above: Analysis of volatile faction, Table 2). Due to its high volatility, this compound will not have a significant impact on the perception of consumers of heat treated products.
