# Supplementary material for: Impact of gaseous ozone treatment of fish carcasses (Gadus morhua) on the microbiological load and their quality
Source: PLoS One. 2025 Aug 18;20(8):e0327866. doi: 10.1371/journal.pone.0327866 (PMC12360513; doi:10.1371/journal.pone.0327866)
Supplement: Attachment — Submitted filename: response_to_the_reviewer_PONE-D-24-48297R1_auresp_3.docx [file pone.0327866.s007.docx]

Note from Editor:

PONE-D-24-48297R2

Impact of gaseous ozone treatment of fish carcasses (Gadus morhua) on the microbiological load and their quality.

PLOS ONE

Dear Dr. Antos,

Thank you for submitting your manuscript to PLOS ONE. After careful consideration, we feel that it has merit but does not fully meet PLOS ONE’s publication criteria as it currently stands. Therefore, we invite you to submit a revised version of the manuscript that addresses the points raised during the review process.

=============
Request from the Editorial Office:
In line with the principles expressed in the Declaration of Helsinki, we expect all research involving human participants and/or medical data to have been approved by the authors' Institutional Review Board (IRB) or by equivalent ethics committee(s). If the need for ethical approval is waived, this should be formally confirmed by a suitable committee generally before the start of the study.

As the requirements of journals and institutions are becoming stricter, approval from an independent ethics committee is becoming the norm for all research studies involving human participants and/or medical information independently of how low the risks are. Please note that we reserve the right to reject any submission that does not meet these standards, which in some cases are more stringent than local ethical standards.

Therefore before we can proceed further, we would require your institutional ethics committee to formally confirm that ethical approval was not needed in this case. Please note that we do not accept retrospective ethics approval. (If approval is needed, this should have been done before the start of the study.) Please include a copy of the letter from the ethics committee as an "Other" file.

We hope you understand the reasons behind this request and look forward to hearing from you.

**Answer for the Editor:**

The procedure for obtaining ethical approval for research involving human subjects requires extensive paperwork and is both costly and time-consuming. Therefore, the authors have decided to remove the section describing human subjects research from the manuscript. We hope the revised version will still be considered suitable for publication

**Action taken:**

The description of sensory analysis and its discussion have been removed from the manuscript.

**~~Method of sensory analysis~~**

~~As a part of the conducted research, the chemical investigations were supplemented only by a preliminary sensory analysis. For this purpose, the samples that were intended to be subjected to the SPME analysis were previously subjected to a preliminary sensory analysis by the authors of the manuscript, who described the sensory experiences while smelling the sample. The authors were trained to be able to participate in sensory analysis of food products and therefore also ozonated fish matrix. All of them volunteered as the subjects for sensory analysis of fish matrix exposed to ozonation. Therefore, no additional approval of ethic committee was asked for.~~

**~~Discussion on sensory analysis~~**

~~Finally a preliminary sensory analysis of the fish tissue performed without heat treatment indicated the presence of foreign odour notes, which are typical of carbonyl products of fatty acid decomposition. Furthermore, in the volatile fraction, a representative of these compounds was identified, i.e., reported above pentadecanal (see section above:~~ ~~Analysis of volatile faction, Table 2). Due to its high volatility, this compound will not have a significant impact on the perception of consumers of heat treated products.~~
